# Inpatient vs. Outpatient: A Systematic Review of Information Needs throughout the Heart Failure Patient Journey

**DOI:** 10.3390/jcm13041085

**Published:** 2024-02-14

**Authors:** Lisa M. Cotie, Maureen Pakosh, Gabriela Lima de Melo Ghisi

**Affiliations:** 1 KITE Research Institute, Toronto Rehabilitation Institute, University Health Network, Toronto, ON M4G 2V6, Canada; lisa.cotie@uhn.ca (L.M.C.);; 2 Department of Physical Therapy, Temerty Faculty of Medicine, University of Toronto, Toronto, ON M5S 3H2, Canada

**Keywords:** heart failure, patient education, information needs, tailored interventions, chronic disease management, inpatient setting, outpatient setting

## Abstract

The objective of this systematic review was to identify and describe information needs for individuals with heart failure (HF) throughout their patient journey. Six databases were searched (APA PsycINFO, CINAHL Ultimate, Embase, Emcare Nursing, Medline ALL, and Web of Science Core Collection) from inception to February 2023. Search strategies were developed utilizing the PICO framework. Potential studies of any methodological design were considered for inclusion through a snowball hand search. Data from the included articles were extracted by a reviewer, and the extraction accuracy was independently cross-checked by another author. Quality appraisal was assessed using the Mixed-Methods Appraisal Tool. A narrative synthesis was used to analyze all the outcomes according to the Synthesis Without Meta-analysis reporting guidelines. Twenty-five studies (15 quantitative and 10 qualitative) were included. Socioeconomic, cultural, and demographic factors influencing information needs were considered. The top three information needs for outpatients included general HF information, signs and symptoms and disease management strategies. For inpatients, medications, risk factors, and general HF were reported as the top needs. These divergent needs emphasize the importance of tailored education at different stages. Additionally, the review identified gaps in global representation, with limited studies from Africa and South America, underscoring the need for inclusive research. The findings caution against overgeneralization due to varied reporting methods. Practical implications call for culturally sensitive interventions to address nuanced HF patients’ needs, while future research must prioritize standardized reporting, consider diverse patient journey timepoints, and minimize biases for enhanced reliability and applicability.

## 1. Introduction

Heart failure (HF) is a medical condition characterized by the diminished ability of the heart to pump or fill with blood, resulting in a reduced cardiac output due to structural and/or functional abnormalities [1]. The global prevalence of HF in the general adult population ranges from 1 to 3%, a figure anticipated to rise with ongoing advancements in diagnostic methods and treatment modalities [1]. HF manifests in various phenotypes, such as HF with a preserved ejection fraction (HFpEF) and HF with a reduced ejection fraction (HFrEF) [2]. While improvements in treatments are expected to stabilize or decrease the prevalence of HFrEF, there is a steady increase in the occurrence of HFpEF [1]. The 5-year risk of HF-related mortality remains substantial, reaching approximately 75% [3]. The economic burden associated with HF is significant, with an estimated annual healthcare cost of around $36,000 CAD per patient in developed countries [1]. A substantial portion of these costs arise from inpatient care and the occurrence of rehospitalization [4].

Patient education has a pivotal role in the care of those living with HF [5]. Management programs for HF, incorporating education as a key element, prove successful in enhancing self-care practices, thereby leading to a decrease in hospital readmission rates [6,7,8]. Moreover, these programs have the potential to lower the likelihood of HF-related hospitalization by 20% [9]. A systematic review of 35 self-management education studies, involving 7413 congestive heart failure patients, demonstrated that structured educational interventions positively impacted disease-related knowledge, self-monitoring, medication adherence, time to hospitalization, and days spent in the hospital [10].

Educating HF patients before hospital discharge has been shown to reduce readmissions, assist with self-identification of problems earlier, and promote improved self-care [11]. These initiatives are particularly significant for individuals living with chronic HF, contributing to a minimized need for re-hospitalizations and an overall improvement in quality of life [12]. Addressing the challenge of hospital admissions and readmissions through improved self-management is crucial for the long-term well-being of HF patients [10]. It should be a primary focus for clinicians, researchers, and other healthcare stakeholders, aiming to alleviate the burden on the healthcare system while enhancing patient outcomes [2].

Research indicates a disparity in educational priorities between patients and their healthcare providers [13]. While both groups recognize the significance of information related to medications and signs and symptoms of HF, clinicians tend to assign greater importance to lifestyle factors, such as diet and weight management, compared to patients [14]. To bridge this gap, healthcare providers must gain a deeper understanding of the learning needs of HF patients and tailor education to be more patient-centered [14,15].

According to adult learning principles, it is crucial to recognize that adults learn best when the content is personally meaningful to them [16]. Therefore, understanding the specific information required from the patient’s perspective becomes paramount. Notably, it is essential to acknowledge the differences between patients admitted to the hospital with HF and those receiving outpatient care. Inpatients often present with more severe illness, multiple comorbidities, and higher event rates [17]. Consequently, the information needs of HF patients evolve throughout their healthcare journey, emphasizing the importance of adapting education to different phases of patient care. Hence, the objective of this systematic review was to identify and describe information needs for individuals living with HF throughout their patient journey. The findings from this review may offer valuable insights into structuring educational interventions that can effectively cater to the varied needs of HF patients, potentially adapting to different settings based on patients’ circumstances, whether in inpatient or outpatient care.

## 2. Materials and Methods

The reporting of this study followed the Preferred Reporting Items for Systematic Reviews and Meta-Analyses (PRISMA) checklist [18]. This systematic review was prospectively registered at the Open Science Framework (https://osf.io/k2egt/ (accessed on 4 October 2023)). Ethical review and approval were waived for this study because this is a systematic review that did not include participants.

### 2.1. Eligibility Criteria

This review included studies that examined the information requirements reported by individuals with HF, utilizing questionnaires tailored to assess information needs, qualitative data collected via interviews and focus groups, or any patient-reported outcome measures directly evaluating the information sought by HF patients. Information needs reported by family members, caregivers, and/or healthcare providers were not considered for this review. Studies using any methodological design were considered for inclusion in this review (i.e., quantitative, qualitative, and mixed methods). Additionally, narrative, systematic, and scoping reviews were examined as a possible source of additional primary studies. The selection criteria did not impose any restrictions based on language for studies to be eligible for inclusion.

### 2.2. Information Sources and Search Strategy

Six academic databases were searched, including APA PsycINFO, CINAHL Ultimate, Embase, Emcare Nursing, Medline ALL, and Web of Science Core Collection (Inception to 16 February 2023). The search strategies were developed in collaboration with an Information Specialist (MP) utilizing the PICO (i.e., Population, Intervention, Comparator, Outcome) framework, subject headings as appropriate for each database, and free-text terms relevant to the topical concepts. The full Medline search strategy is available in the Supplementary Material File S1.

### 2.3. Selection Process, Data Collection Process and Data Items

Following the removal of duplicates in Covidence (Covidence systematic review software 2023, Veritas Health Innovation, Melbourne, Australia, available at www.covidence.org), two independent reviewers (LMV and GLMG) screened titles and abstracts of all the records. Full texts of the remaining citations were then independently reviewed to determine whether they met the outlined eligibility criteria (LMC and GLMG). For all stages of the screening, if disagreements between the reviewers existed, consensus was achieved through discussion. The reviewers were not blinded to the authors or journals of the papers throughout the screening process.

One researcher (LMC) independently extracted all relevant data related to the study characteristics (authors, titles, year of publication, journal, country, language, study design, sample size), description of the population (i.e., diagnosis, nationality, sex, mean age/age range), stage of the HF journey (i.e., inpatient, outpatient), and relevant educational needs into an Excel spreadsheet. All extracted data were verified by a second researcher (GLMG). Discrepancies were resolved by consensus.

### 2.4. Quality Assessment

The quality of the articles was then assessed using the Mixed-Methods Appraisal Tool (MMAT) [19]. To use the MMAT, the study design (i.e., qualitative, quantitative RCT, quantitative non-randomized, quantitative descriptive, and mixed methods) of each included citation is determined and five criteria are assessed based on the type of study. Each of the five items is rated as present (yes), not present (no), or indeterminant (unable to tell) [19]. LMC and GLMG separately scored each included study and compared scores upon completion.

### 2.5. Synthesis Methods

The results were analyzed accordingly to the Synthesis Without Meta-analysis (SWiM) reporting guidelines [20]. A narrative synthesis was used to analyze all the outcomes that could not be meta-analyzed. Information needs were considered across the patient journey and ultimately were categorized into inpatient vs. outpatient needs. Only those identified as the primary needs (i.e., endorsed by the majority of participants, exceeding 50%) were incorporated into this review. Due to the nature of the data included in this review, a formal meta-analysis was not possible.

## 3. Results

**Figure 1:**
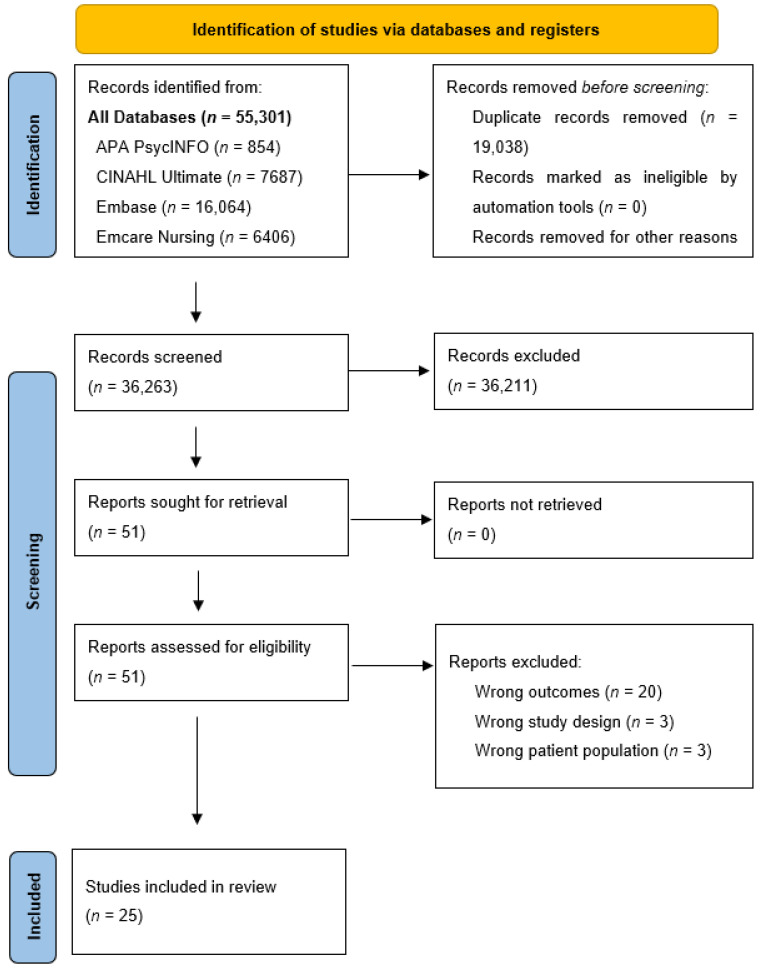
PRISMA flow diagram.

The initial database search yielded 55,301 records. After the initial and secondary screenings, a total of 51 full-text articles were assessed for eligibility. Overall, 25 studies were included in the review. This study selection process is illustrated in the PRISMA flow diagram (Figure 1).

### 3.1. Characteristics of the Included Studies

Table 1 summarizes the characteristics of the 25 included studies, published between 1994 and 2022. Studies were conducted in Europe (*n* = 8) [21,22,23,24,25,26,27,28], North America (*n* = 7) [29,30,31,32,33,34,35], Asia (*n* = 6) [14,36,37,38,39,40], Australia (*n* = 3) [41,42,43], and Africa (*n* = 1) [44]. Twenty three of the studies were published in English [14,21,22,23,24,25,28,29,30,31,32,33,34,35,36,37,38,39,40,41,42,43,44], one in French [26], and one in Italian [27]. In total, 56% percent of the studies included patients from an outpatient perspective [14,21,22,23,25,27,28,30,33,35,36,37,39,43], 32% of studies were from an inpatient perspective [26,29,31,32,34,38,40,42], and 12% of studies included both inpatient and outpatient stages of the patient journey [24,41,44]. Overall, 15 of the 25 studies had a quantitative descriptive design [14,21,22,23,28,29,30,31,32,34,36,37,38,40,41], while the remaining 10 were qualitative in nature [24,25,26,27,33,35,39,42,43,44].

The sample sizes of the included studies ranged from 10 [25] to 347 [38] (median = 50), with a total of 1687 heart failure patients across all studies. Fourteen studies provided information related to the New York Heart Association (NYHA) functional classification (I–IV) [14,22,24,28,29,34,36,37,38,39,40,41,42,44], which included participants from all classifications. Of the 1687 participants, 38% were women. Age was presented differently across studies, including range, mean ± SD, and percent above a specified cut-off. All age data can be found in Table 1.

Table 1 also shows the quality appraisal of the included studies. In total, 10 of the 25 studies had “yes” responses for all five categories (i.e., score of 5/5, which indicates “good” quality) [14,24,27,34,35,37,38,40,42,44], 10 had one “no” or “can’t tell” responses [22,25,26,28,29,30,31,32,41,43] and the remaining 5 had two or more “no” or “can’t tell” responses out of five, indicating low quality [21,23,33,36,39]. No studies were excluded due to low quality.

In total, 11 [14,29,30,31,32,34,36,37,38,40,41] of the 25 included studies used a variety of adaptations of the cardiac patient learning needs inventory (CPLNI) [45] to measure patients HF learning needs. The CPLNI asks users to rank the importance, from 1 (not important) to 5 (very important) of having knowledge about 43 items categorized into 8 domains. These domains include general disease information, anatomy and physiology, psychological factors, risk factors, medication information, diet information, physical activity and other pertinent information. Ten studies used interviews or focus groups [24,25,26,27,33,35,39,42,43,44], four used questionnaires design specifically for the study (two used Likert scales, one multiple choice and one used a question prompt list) [21,23,26,43], and the remaining studies used other surveys [22,28].

### 3.2. Information Needs of Heart Failure Patients in Outpatient Settings

Fourteen of the twenty-five included studies had HF patients that were not admitted to hospital at the time of the study [14,21,22,23,25,27,28,30,33,35,36,37,39,43]. Among the various learning needs of these patients, the top three included general HF information (e.g., definition of HF, how it is diagnosed, prognosis), signs and symptoms, and disease management strategies. Specifically, 6 of the 14 “outpatient” studies listed general information related to HF [21,23,25,27,36,43] and signs and symptoms of HF [14,27,28,30,33,37] among the top three learning needs. Five of the studies listed various disease management strategies (i.e., behaviour/lifestyle, daily salt intake, fluid intake/weight management) as a top learning need rated by the patients [21,27,33,36,39]. Two studies focused on information needs specifically related to sexuality after HF diagnosis. Patients in these studies described information related to communication with their partner, relaxation and comfort in their relationship and sexual environment, and finally, the role of HF symptoms in and during sexual activity as the most important [22,28].

### 3.3. Information Needs of Heart Failure Patients in Inpatient Settings

Eight of the studies had patients admitted to the hospital at the time of data collection [26,29,31,32,34,38,40,42]. The top information needs reported in these studies were related to medications, risk factors, and general HF (Table 2). Seventy-five percent of the studies had medication information in the top three learning needs of patients [26,29,31,32,34,42]. Fifty percent of the eight inpatient studies had general HF information [34,39,40,42] and risk factors [31,32,38,40] ranked as top learning needs.

**Figure 2:**
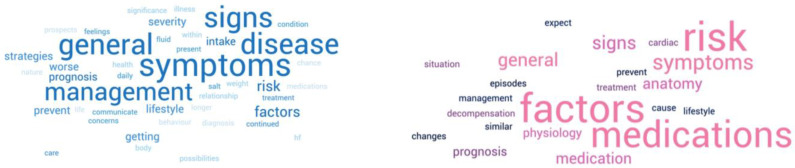
Top information needs in outpatients (**blue**) and inpatients (**pink**).

Three studies included both in- and outpatients as participants [24,41,44]. Signs and symptoms of HF were amongst the highest ranked learning needs in two of the three studies [24,41], while prognosis [41], risk factors [41] and disease management (lifestyle changes) [44] were other important educational topics listed in these studies. The top information needs in outpatients and inpatients are illustrated as a word cloud in Figure 2.

## 4. Discussion

This review identified 25 studies aimed at characterizing the information needs of individuals throughout their patient journey with HF. Employing a patient journey mapping approach [46], we categorized the outcomes to delineate information needs for both inpatients and outpatients. Patients in the outpatient stage prioritized general HF information, signs and symptoms, and disease management strategies as their top three information needs. Conversely, those in the inpatient stage of their HF journey highlighted information about medications, risk factors, and general HF information as their primary priorities. Three studies encompassed both in- and outpatients, identifying signs and symptoms of HF, prognosis, risk factors, and disease management (lifestyle changes) as crucial information needs. Additionally, two studies focused on information needs related to sexuality, revealing that patients emphasized communication with their partner, comfort in their relationship and environment, and the impact of sexual activity on their symptoms as top priorities.

It is unsurprising that individuals in the outpatient stage of HF prioritized general HF information, signs and symptoms, and disease management as their top informational needs. When patients are at home, without the constant care and assistance provided by hospital staff, the onus of managing their disease falls upon themselves [47]. Consequently, a crucial aspect of their self-management revolves around a comprehensive understanding of the signs and symptoms associated with HF, particularly those that may signify serious conditions leading to potential readmission to the hospital [48]. In terms of disease management, patients expressed a keen interest in obtaining information related to their daily salt and fluid intake. Additionally, they sought guidance on the significance of monitoring their daily body weight, which is aligned with practical management recommendations for this patient population [47]. This emphasis on disease management underscores the critical role patients play in their own care, necessitating a thorough comprehension of key aspects to enhance their ability to manage HF effectively within a home environment.

Interestingly, there was a notable trend in which information pertaining to physical activity restrictions, psychological factors, and general food intake ranked as the least important among study participants. It is crucial to highlight that cardiac rehabilitation (CR) stands as a class IA recommendation for HF patients [2,49], as its participation has been demonstrated to enhance functional capacity [50,51], improve quality of life [50,51], and significantly reduce the risk of hospital readmission [52], particularly for those with HFrEF [51,53]. Despite these well-established benefits, unfortunately, patients with HF are largely excluded from CR programs [54]. Regrettably, studies reveal a concerning gap in CR referrals for HF patients, with as few as one quarter of eligible individuals receiving such referrals upon discharge following hospitalization for HF and less than 5% of those referred ultimately participating in CR [55]. This dearth in referrals might elucidate why HF outpatients ranked information related to physical activity, psychological factors, dietary considerations beyond salt intake, as well as lifestyle factors like alcohol intake and smoking as low priorities in their informational needs. This underscores the need for increased awareness and integration of CR into the overall care plan for HF patients, considering its proven benefits for both functional outcomes and the reduction in hospital readmissions.

Individuals who were inpatients for these studies ranked information about medications, risk factors and general disease information as their highest learning needs. Specifically, inpatients displayed a keen interest in understanding the potential side effects of medications and how to address issues that might arise in connection with their medication regimen. This is important as studies have shown the implementation of guideline-directed medical therapy remains below optimal levels [56]. Research shows that clinicians consistently underscore the significance of medications as a crucial component of chronic disease management for individuals with HF [31]. Frequently, hospital readmissions and clinic visits are strategically scheduled around adjustments, additions, or removals to the medication regimen, aiming to better control symptoms [57]. It is possible that when patients receive information that directly influences their well-being, improves their symptoms, and reduces the likelihood of hospitalization, they exhibit a heightened motivation to retain such knowledge [31]. As patients prepare for discharge, understanding the risk factors that may exacerbate their condition becomes paramount [34]. Notably, individuals with HF who are inpatients generally exhibit a higher level of illness severity and experience higher event rates compared to their outpatient counterparts [17]. Consequently, the information needs for these distinct groups diverge, necessitating tailored educational approaches to address the unique challenges and requirements of inpatient and outpatient populations.

It is noteworthy to highlight that 21 of the 25 included studies were published before 2020. Assessing the information needs of patients in the current time period is of paramount importance, especially considering the significant changes that have occurred in the healthcare landscape with the advent of the COVID-19 pandemic [58]. The unprecedented challenges posed by the pandemic have introduced new dimensions to the healthcare system, impacting patients with HF who also contracted the COVID-19 virus. The coexistence of HF and COVID-19 may introduce pharmacological and symptomatic implications that need careful consideration in understanding the holistic needs of these patients [58,59]. Additionally, as knowledge and treatment options improve over time, understanding the needs of patients with respect to education is important to understand. Regularly updated data understanding the information needs of HF patients are necessary to ensure the most current information is shared with patients.

There are many factors related to HF that can influence sexuality, including pharmacological treatments, physiological aspects, and the broader psychological and emotional ramifications of receiving a diagnosis of HF [60,61,62]. Research indicates that over half of patients with HF encounter sexual challenges, ranging from difficulties to a complete cessation of sexual activity due to their diagnosis [22,28]. However, reassuringly, evidence suggests that despite a decline or cessation in sexual activity, there appears to be little to no impact on other aspects of relationships [22]. Historically, sexual counseling has been surrounded by stigma, perceived as shameful, embarrassing, and anxiety-provoking [63,64]. Additionally, the topic of sexuality is taboo in many cultures [63,65,66], necessitating a culturally sensitive approach when providing information to patients. The results from this systematic review highlight the need for sexual information by HF individuals. Research suggests that sexual information should be discussed at a variety of times through the patient journey and thus falls to the responsibility of the entire clinical team [22,28]. A better understanding of the specific information needs of HF patients can help guide these discussions [22,28].

When identifying the information needs of HF patients, it is crucial to take into account variables such as sex and gender, age, socioeconomic status, and cultural differences [5] Each of these factors is likely to exert a substantial influence on the unique information requirements of patients. Socioeconomic status, for example, can play a pivotal role in determining patients’ access to various treatment options, including pharmacotherapy and CR [67]. Cultural considerations are equally significant, encompassing the need to comprehend and adapt information related to food intake, treatment options, and sensitive topics such as sexuality and relationships [68]. Moreover, sex, gender, and age are important factors that may significantly shape the information needs of HF patients. Notably, four studies included in this review reported differences in information needs between men and women, underscoring the importance of considering these demographic variables in tailoring educational strategies [21,22,28,30].

The results from this review should be interpreted with caution. One notable limitation arises from the diverse methods employed to describe patient characteristics across the included studies. This variability in reporting hindered our ability to create a more nuanced and detailed mapping of the patient’s journey. While we presented results from the distinct inpatient and outpatient stages of the HF journey, it is essential to acknowledge that information needs may vary at other critical junctures in a patient’s trajectory, such as the time of diagnosis, during treatment (including device interventions), or in the context of end-of-life care. Future studies are needed—given that only four included studies are dated during or after 2020—and should endeavor to provide a more comprehensive understanding of information needs across each stage of the HF patient journey to capture potential changes over time. Another limitation stems from the geographical scope of the studies, with a notable absence of research conducted in certain regions of the world, particularly Africa and South America. Consequently, the generalizability of the results may be constrained, and caution should be exercised when applying these findings globally. Cultural differences may lead to differences in information needs between regions. A comprehensive understanding of information needs in HF necessitates a global perspective that considers the influence of cultural nuances and regional disparities.

Furthermore, it is important to recognize that our analysis was strictly descriptive, and a formal assessment of potential bias was not conducted.

In conclusion, this review offers valuable insights into the information needs of individuals navigating the complex journey of HF, and what clinicians must consider to ensure they understand patients’ information needs at each stage. The main information needs vary by different stages of HF, with patients in the outpatient stage prioritizing signs and symptoms and disease management strategies, while those in the inpatient stage prioritize information about medications and risk factors. Both groups are keen to learn general information about their condition, which highlights the need to include education throughout their patient journey. It is likely these differences are more granular than the outpatient vs. inpatient stages of the patient journey, and therefore it is the responsibility of clinicians to assess patient information needs multiple times throughout their HF journey. Providing important information to patients can help to decrease hospital readmissions, ultimately leading to better patient outcomes and decreased healthcare costs.

The findings of this review bear significant practical implications for healthcare providers, policymakers, and researchers. Firstly, the recognition of varied information needs throughout the HF journey underscores the importance of tailoring patient education interventions to meet specific requirements and enhance HF management. This personalized approach holds potential to enhance self-management, diminish hospital readmissions, and improve overall patient outcomes [69,70]. Additionally, the review underscores the importance of cultural sensitivity, highlighting the necessity for healthcare professionals to tailor information delivery to diverse cultural norms. Integrating sexual counseling and addressing taboos surrounding certain topics can foster a more supportive and patient-centered care environment. Moving forward, there is a need for discussions regarding future directions for improving HF management, considering implementation science studies and/or pragmatic trials to bridge the gap between guidelines and real-world practices.

The limited global representation in the current body of research signals a call for more inclusive studies. Understanding information needs across cultures and regions is essential for providing equitable care. Policymakers should advocate for and support research initiatives that span the globe, fostering a comprehensive understanding of HF patients’ information needs. In summary, the practical implications involve adopting a patient-centered, culturally sensitive, and globally inclusive approach to address the multifaceted information needs of heart failure patients. This, in turn, can lead to more effective healthcare strategies, improved patient outcomes, and a higher quality of life for individuals grappling with this chronic condition.

## Figures and Tables

**Table 1:** Study characteristics.

Author Year Country Language	Setting In- vs. Out-Patient	Study Design Quality Appraisal (/5) *	Participant Characteristics			
			Sample Size	NYHA Classifications	Females (*n*) or (%)	Age (*n*) or (Range)
Andersson, L. et al. [21] 2019 Sweden English	6 primary healthcare centers Outpatients	Quantitative descriptive 3/5	192	Not reported	*n* = 97 51%	48–96 (mean 80.3 ± 9.2)
Ashour, A. et al. [36] 2020 Jordan English	Outpatient department of 2 government hospitals Outpatients	Quantitative descriptive 3/5	67	I = 7.5% II = 11.9% III = 19.4% IV = 61.2%	*n* = 29 43%	60 years or older (71.6%)
Baert, A. et al. [22] 2019 Italy and Belgium English	4 hospitals Outpatients	Quantitative descriptive 4/5	77	I = 13.85% II = 72.3% III = 12.3%	*n* = 20 27%	62.3 (SD = 10.6)
Banerjee, P. et al. [23] 2010 United Kingdom English	Patients identified via hospital database Outpatients	Quantitative descriptive 3/5	90	Not reported	*n* = 29 32%	43–87 (median 71)
Boyde, M. et al. [41] 2009 Australia English	Large tertiary referral hospital In- and outpatients	Quantitative descriptive 4/5	55	I = 9% II = 44% III = 44% IV = 3%	*n* = 17 31%	33–83 years—mean 64.25 (SD = 13.21)
Buetow, S. et al. [43] 2001 New Zealand English	Large primary care organization Outpatients	Qualitative 4/5	62	Not reported	*n* = 26 42%	75 years (SD = 11)
Chan, A. et al. [29] 2003 Canada England	1 hospital Inpatients	Quantitative descriptive 4/5	34	II = 17.6% III = 20.6% IV = 38.2%	*n* = 6 18%	54.8 (14.88 SD), range 26 to 83
Clark, J. et al. [30] 2004 USA English	Home care and outpatient HF clinic Outpatients	Quantitative descriptive 4/5	33	Not reported	*n* = 17 51%	49–92 years, mean = 75.7 (SD = 10.56)
Frattini, E. et al. [31] 1998 Canada English	Hospital Inpatients	Quantitative descriptive 4/5	50	Not reported	*n* = 15 30%	56–75 years
Hagenhoff, B. et al. [32] 2008 United Kingdom English	Hospital Inpatients	Quantitative descriptive 4/5	30	Not reported	*n* = 10 33%	68 years (SD = 12.81) (range: 38–87)
Harding, R. et al. [24] 2008 United Kingdom English	Outpatient clinic and hospital wards In- and outpatients	Qualitative 5/5	20	III = 70% III = 10% IV = 20%	*n* = 4 20%	69 years (SD = 10.6)
Ivynian, S. et al. [42] 2020 Australian England	Inpatient lists from the cardiothoracic ward at a teaching hospital Inpatients	Qualitative 5/5	15	II = 47% III = 53%	*n* = 5 33%	55 years (median), IQR 52.69
Jenkins, H. et al. [33] 2022 USA English	Outpatient at a cardiology clinic Outpatients	Qualitative 3/5	30	Not reported	*n* = 11 37%	60.3 (13.9) years (mean) for accompanied patients; 61.14 (17.2) for unaccompanied
Kim, S. et al. [37] 2013 Korea English	Cardiac outpatient clinic Outpatients	Quantitative descriptive 5/5	121	I = 49.1% II = 38.8% III = 12.1%	*n* = 41 34%	19–88 years, 68% were >60 years of age, 19% were 50–59 and 13% were <50
Kimani, K. et al. [44] 2018 Kenya English	Hospital In- and outpatients	Qualitative 5/5	18	III = 56% IV = 44%	*n* = 10 56%	Not reported
Kristiansen, A. et al. [25] 2017 Denmark English	Hospital Outpatients	Qualitative 4/5	10	Not reported	*n* = 2 20%	47–78 years
Lhermitte, C. et al. [26] 2019 France French	Hospital Inpatients	Qualitative 4/5	15	Not reported	*n* = 6 40%	80.1 years
Min, D. et al. [14] 2020 Korea English	2 large tertiary medical centers Outpatients	Quantitative descriptive 5/5	100	I = 24% II = 25% III = 35% IV = 16%	*n* = 58 58%	72.15 years (range 44–94)
Ong, S. et al. [40] 2018 Singapore English	Acute tertiary hospital Inpatients	Quantitative descriptive 5/5	97	I = 19.6% II = 30.9% III = 34% IV = 9.3%	*n* = 28 30%	60.84 (SD = 11.34), range 32–89
Pianese, M. et al. [27] 2011 Italy Italian	Hospital clinic Outpatients	Qualitative 5/5	50	Not reported	*n* = 23 46%	77.68 years
vanDriel, A. et al. [28] 2014 Belgium and Netherlands England	3 hospitals Outpatients	Quantitative descriptive 4/5	52	I = 15% II = 42% III = 23% IV = 15%	*n* = 11 21%	65 years
Wehby, D. et al. [34] 1999 USA English	2 Hospitals Inpatients	Quantitative descriptive 5/5	84	II = 30% III = 56% IV = 14%	*n* = 45 54%	71.8 (SD = 12.86), range 33–92 years
Williams, M. [35] 2019 USA English	Follow-up visit after hospital discharge Outpatients	Qualitative 5/5	12	Not reported	*n* = 2 17%	57.1
Yu, M. et al. [38] 2012 China English	3 Hospitals Inpatients	Quantitative descriptive 5/5	347	I = 10% II = 55% III = 25% IV = 10%	*n* = 123 35%	64 (SD = 13)
Yu, M. et al. [39] 2016 China English	Cardiovascular department of a university-affiliated hospital Outpatients	Qualitative 2/5	26	II = 38.5% III = 34.6% IV = 26.9%	*n* = 11 42%	58.62 (SD = 11.09)

* Mixed Methods Appraisal Tool.

**Table 2:** Top 3 ranked information needs reported in the included studies (*n* = 25).

Setting	Reference	Top 3 Information Needs
Outpatient	Andersson et al., 2019 [21]	General HF knowledge Medications Continued care and treatment
	Ashour et al., 2020 [36]	Daily salt intake Fluid intake Present condition
	Baert et al., 2019 [22]	How to communicate feelings, concerns, and possibilities within their relationship NR NR
	Banerjee et al., 2010 [23]	General HF knowledge NR NR
	Buetow et al., 2001 [43]	General HF knowledge NR NR
	Clark et al., 2004 [30]	Signs and symptoms Medications Prognosis
	Jenkins et al., 2022 [33]	Severity of illness Health body weight Signs of HF getting worse/how to prevent it getting worse
	Kristiansen et al., 2017 [25]	Significance of diagnosis Prospects Chance of a longer life
	Min et al., 2020 [14]	Medications Signs and symptoms Risk factors
	Pianese et al., 2011 [27]	Signs and symptoms Behaviour/lifestyle Nature, causes and severity of disease
	vanDriel et al., 2014 [28]	Relationships Relaxation Symptoms
	Williams et al., 2019 [35]	Discharge preparation Coping strategies Community education
	Yu et al., 2016 [39]	Self-management Prevention Strategies for controlling the disease
Inpatient	Chan et al., 2003 [29]	Medications Anatomy Treatment
	Frattini et al., 1998 [31]	Medication Risk factors Anatomy and physiology
	Hagenhoff et al., 1994 [32]	Medications Anatomy and physiology Risk factors
	Ivynian et al., 2020 [42]	General HF knowledge Medication Signs and symptoms
	Kim et al., 2013 [37]	Signs and symptoms Medications Risk factors
	Lhermitte et al., 2019 [26]	Situation of cardiac decompensation Medication NR
	Ong et al., 2018 [40]	Signs and symptoms Risk factors General HF knowledge
	Wehby et al., 1999 [34]	Medications Signs and symptoms General HF knowledge
	Yu et al., 2012 [38]	Risk factors Prognosis General HF knowledge
Both outpatient and inpatient	Boyde et al., 2009 [41]	Signs and symptoms Prognosis Risk factors
	Harding et al., 2008 [24]	Symptoms (cause, management, what to expect) NR NR
	Kimani et al., 2018 [44]	Lifestyle changes to make to prevent similar episodes NR NR

HF: heart failure; NR: not reported.

## Data Availability

No new data were created or analyzed in this study. Data sharing is not applicable to this article.

## Supplementary Materials

The following supporting information can be downloaded at: https://www.mdpi.com/article/10.3390/jcm13041085/s1, File S1: Medline Search Strategy.

## References

[1] Savarese G., Becher P.M., Lund L.H., Seferovic P., Rosano G.M.C., Coats A.J.S. (2023). Global burden of heart failure: A comprehensive and updated review of epidemiology. Cardiovasc. Res..

[2] Ponikowski P., Voors A.A., Anker S.D., Bueno H., Cleland J.G., Coats A.J., Falk V., González-Juanatey J.R., Harjola V.P., Jankowska E.A. (2016). 2016 ESC Guidelines for the diagnosis and treatment of acute and chronic heart failure: The Task Force for the diagnosis and treatment of acute and chronic heart failure of the European Society of Cardiology (ESC)Developed with the special contribution of the Heart Failure Association (HFA) of the ESC. Eur. Heart J..

[3] Roger V.L. (2021). Epidemiology of Heart Failure. Circ. Res..

[4] Cook C., Cole G., Asaria P., Jabbour R., Francis D.P. (2014). The annual global economic burden of heart failure. Int. J. Cardiol..

[5] Strömberg A. (2005). The crucial role of patient education in heart failure. Eur. J. Heart Fail..

[6] McAlister F.A., Stewart S., Ferrua S., McMurray J.J. (2004). Multidisciplinary strategies for the management of heart failure patients at high risk for admission: A systematic review of randomized trials. J. Am. Coll. Cardiol..

[7] Strömberg A., Mårtensson J., Fridlund B., Dahlström U. (2001). Nurse-led heart failure clinics in Sweden. Eur. J. Heart Fail..

[8] de Loor S., Jaarsma T. (2002). Nurse-managed heart failure programmes in the Netherlands. Eur. J. Cardiovasc. Nurs..

[9] Jonkman N.H., Westland H., Groenwold R.H., Ågren S., Atienza F., Blue L., Bruggink-André de la Porte P.W., DeWalt D.A., Hebert P.L., Heisler M. (2016). Do Self-Management Interventions Work in Patients with Heart Failure? An Individual Patient Data Meta-Analysis. Circulation.

[10] Boren S.A., Wakefield B.J., Gunlock T.L., Wakefield D.S. (2009). Heart failure self-management education: A systematic review of the evidence. Int. J. Evid. Based Healthc..

[11] Paul S. (2008). Hospital discharge education for patients with heart failure: What really works and what is the evidence?. Crit. Care Nurse.

[12] Albert N.M. (2016). A systematic review of transitional-care strategies to reduce rehospitalization in patients with heart failure. Heart Lung.

[13] Kennedy B.M., Rehman M., Johnson W.D., Magee M.B., Leonard R., Katzmarzyk P.T. (2017). Healthcare Providers versus Patients’ Understanding of Health Beliefs and Values. Patient Exp. J..

[14] Min D., Park J.S., Choi E.Y., Ahn J.A. (2020). Comparison of learning needs priorities between healthcare providers and patients with heart failure. PLoS ONE.

[15] McDonald M.A., Ashley E.A., Fedak P.W., Hawkins N., Januzzi J.L., McMurray J.J., Parikh V.N., Rao V., Svystonyuk D., Teerlink J.R. (2017). Mind the Gap: Current Challenges and Future State of Heart Failure Care. Can. J. Cardiol..

[16] Palis A.G., Quiros P.A. (2014). Adult learning principles and presentation pearls. Middle East. Afr. J. Ophthalmol..

[17] Ferreira J.P., Metra M., Mordi I., Gregson J., ter Maaten J.M., Tromp J., Anker S.D., Dickstein K., Hillege H.L., Ng L.L. (2019). Heart failure in the outpatient versus inpatient setting: Findings from the BIOSTAT-CHF study. Eur. J. Heart Fail..

[18] Page M.J., McKenzie J.E., Bossuyt P.M., Boutron I., Hoffmann T.C., Mulrow C.D., Shamseer L., Tetzlaff J.M., Akl E.A., Brennan S.E. (2021). The PRISMA 2020 statement: An updated guideline for reporting systematic reviews. BMJ.

[19] Hong Q.N., Pluye P., Fàbregues S., Bartlett G., Boardman F., Cargo M., Dagenais P., Gagnon M.P., Griffiths F., Nicolau B. (2021). Mixed Methods Appraisal Tool (MMAT), Version 2018 User Guide. BMJ Open.

[20] Campbell M., McKenzie J.E., Sowden A., Katikireddi S.V., Brennan S.E., Ellis S., Hartmann-Boyce J., Ryan R., Shepperd S., Thomas J. (2020). Synthesis without meta-analysis (SWiM) in systematic reviews: Reporting guideline. BMJ.

[21] Andersson L., Nordgren L. (2019). Heart Failure Patients’ Perceptions of Received and Wanted Information: A Cross-Sectional Study. Clin. Nurs. Res..

[22] Baert A., Pardaens S., De Smedt D., Puddu P.E., Ciancarelli M.C., Dawodu A., De Sutter J., De Bacquer D., Clays E. (2019). Sexual Activity in Heart Failure Patients: Information Needs and Association with Health-Related Quality of Life. Int. J. Environ. Res. Public. Health.

[23] Banerjee P., Gill L., Muir V., Nadar S., Raja Y., Goyal D., Koganti S. (2010). Do heart failure patients understand their diagnosis or want to know their prognosis? Heart failure from a patient’s perspective. Clin. Med..

[24] Harding R., Selman L., Beynon T., Hodson F., Coady E., Read C., Walton M., Gibbs L., Higginson I.J. (2008). Meeting the communication and information needs of chronic heart failure patients. J. Pain Symptom Manag..

[25] Kristiansen A.M., Svanholm J.R., Schjødt I., Mølgaard Jensen K., Silén C., Karlgren K. (2017). Patients with heart failure as co-designers of an educational website: Implications for medical education. Int. J. Med. Educ..

[26] Lhermitte C., Viallet A., Rosset E., Godreuil C. (2019). Assessing the educational needs of patients with heart failure. Soins.

[27] Pianese M., De Astis V., Griffo R. (2011). Assessing patients needs in outpatients with advanced heart failure. Monaldi Arch. Chest Dis..

[28] Driel A.G., de Hosson M.J., Gamel C. (2014). Sexuality of patients with chronic heart failure and their spouses and the need for information regarding sexuality. Eur. J. Cardiovasc. Nurs..

[29] Chan A.D., Reid G.J., Farvolden P., Deane M.L., Bisaillon S. (2003). Learning needs of patients with congestive heart failure. Can. J. Cardiol..

[30] Clark J.C., Lan V.M. (2004). Heart failure patient learning needs after hospital discharge. Appl. Nurs. Res..

[31] Frattini E., Lindsay P., Kerr E., Park Y.J. (1998). Learning needs of congestive heart failure patients. Prog. Cardiovasc. Nurs..

[32] Hagenhoff B.D., Feutz C., Conn V.S., Sagehorn K.K., Moranville-Hunziker M. (1994). Patient education needs as reported by congestive heart failure patients and their nurses. J. Adv. Nurs..

[33] Jenkins H.R., Rupert D.J., Etta V., Peinado S., Wolff J.L., Lewis M.A., Chang P., Cené C.W. (2022). Examining Information Needs of Heart Failure Patients and Family Companions Using a Previsit Question Prompt List and Audiotaped Data: Findings from a Pilot Study. J. Card. Fail..

[34] Wehby D., Brenner P.S. (1999). Perceived learning needs of patients with heart failure. Heart Lung.

[35] Williams M.D. (2019). Exploring Education Needs for Heart Failure Patients’ Transition of Care to Home. Doctoral Dissertation.

[36] Ashour A., Al-Rawashdeh S., Alwidyan M., Al-Smadi A., Alshraifeen A. (2020). Perceived Learning Needs of Patients with Heart Failure in Jordan: Perspectives of Patients, Caregivers, and Nurses: A Cross-Sectional Survey. J. Cardiovasc. Nurs..

[37] Kim S.S., Ahn J.A., Kang S.M., Kim G., Lee S. (2013). Learning needs of patients with heart failure a descriptive, exploratory study. J. Clin. Nurs..

[38] Yu M., Chair S.Y., Chan C.W., Li X., Choi K.C. (2012). Perceived learning needs of patients with heart failure in China: A cross-sectional questionnaire survey. Contemp. Nurse.

[39] Yu M.M., Chair S.Y., Chan C.W., Choi K.C. (2016). Information needs of older people with heart failure: Listening to their own voice. J. Geriatr. Cardiol..

[40] Ong S.F., Foong P.P., Seah J.S., Elangovan L., Wang W. (2018). Learning Needs of Hospitalized Patients with Heart Failure in Singapore: A Descriptive Correlational Study. J. Nurs. Res..

[41] Boyde M., Tuckett A., Peters R., Thompson D.R., Turner C., Stewart S. (2009). Learning style and learning needs of heart failure patients (The Need2Know-HF patient study). Eur. J. Cardiovasc. Nurs..

[42] Ivynian S.E., Newton P.J., DiGiacomo M. (2020). Patient preferences for heart failure education and perceptions of patient-provider communication. Scand. J. Caring Sci..

[43] Buetow S.A., Coster G.D. (2001). Do general practice patients with heart failure understand its nature and seriousness, and want improved information?. Patient Educ. Couns..

[44] Kimani K.N., Murray S.A., Grant L. (2018). Multidimensional needs of patients living and dying with heart failure in Kenya: A serial interview study. BMC Palliat. Care.

[45] Gerard P.S., Peterson L.M. (1984). Learning needs of cardiac patients. Cardiovasc. Nurs..

[46] Joseph A.L., Kushniruk A.W., Borycki E.M. (2020). Patient journey mapping: Current practices, challenges and future opportunities in healthcare. Knowl. Manag. E-Learn..

[47] Jaarsma T., Hill L., Bayes-Genis A., La Rocca H.B., Castiello T., Čelutkienė J., Marques-Sule E., Plymen C.M., Piper S.E., Riegel B. (2021). Self-care of heart failure patients: Practical management recommendations from the Heart Failure Association of the European Society of Cardiology. Eur. J. Heart Fail..

[48] Reeder K.M., Ercole P.M., Peek G.M., Smith C.E. (2015). Symptom perceptions and self-care behaviors in patients who self-manage heart failure. J. Cardiovasc. Nurs..

[49] Yancy C.W., Jessup M., Bozkurt B., Butler J., Casey D.E., Colvin M.M., Drazner M.H., Filippatos G.S., Fonarow G.C., Givertz M.M. (2017). 2017 ACC/AHA/HFSA Focused Update of the 2013 ACCF/AHA Guideline for the Management of Heart Failure: A Report of the American College of Cardiology/American Heart Association Task Force on Clinical Practice Guidelines and the Heart Failure Society of America. Circulation.

[50] Taylor R.S., Walker S., Smart N.A., Piepoli M.F., Warren F.C., Ciani O., Whellan D., O’Connor C., Keteyian S.J., Coats A. (2019). Impact of Exercise Rehabilitation on Exercise Capacity and Quality-of-Life in Heart Failure: Individual Participant Meta-Analysis. J. Am. Coll. Cardiol..

[51] Long L., Mordi I.R., Bridges C., A Sagar V., Davies E.J., Coats A.J., Dalal H., Rees K., Singh S.J., Taylor R.S. (2019). Exercise-based cardiac rehabilitation for adults with heart failure. Cochrane Database Syst. Rev..

[52] Kamiya K., Sato Y., Takahashi T., Tsuchihashi-Makaya M., Kotooka N., Ikegame T., Takura T., Yamamoto T., Nagayama M., Goto Y. (2020). Multidisciplinary Cardiac Rehabilitation and Long-Term Prognosis in Patients with Heart Failure. Circ. Heart Fail..

[53] Taylor R.S., Long L., Mordi I.R., Madsen M.T., Davies E.J., Dalal H., Rees K., Singh S.J., Gluud C., Zwisler A.D. (2019). Exercise-Based Rehabilitation for Heart Failure: Cochrane Systematic Review, Meta-Analysis, and Trial Sequential Analysis. JACC Heart Fail..

[54] Park L.G., Schopfer D.W., Zhang N., Shen H., Whooley M.A. (2017). Participation in Cardiac Rehabilitation Among Patients with Heart Failure. J. Card. Fail..

[55] Keshvani N., Subramanian V., Wrobel C.A., Solomon N., Alhanti B., Greene S.J., DeVore A.D., Yancy C.W., Allen L.A., Fonarow G.C. (2023). Patterns of Referral and Postdischarge Utilization of Cardiac Rehabilitation Among Patients Hospitalized with Heart Failure: An Analysis From the GWTG-HF Registry. Circ. Heart Fail..

[56] Canonico M.E., Hsia J., Cannon C.P., Bonaca M.P. (2022). Uptake of Newer Guideline-Directed Therapies in Heart Failure Patients with Diabetes or Chronic Kidney Disease. JACC Heart Fail..

[57] Bülow C., Clausen S.S., Lundh A., Christensen M. (2023). Medication review in hospitalised patients to reduce morbidity and mortality. Cochrane Database Syst. Rev..

[58] Cowie M.R., Mourilhe-Rocha R., Chang H.-Y., Volterrani M., Ban H.N., de Albuquerque D.C., Chung E., Fonseca C., Lopatin Y., Serrano J.A.M. (2022). The impact of the COVID-19 pandemic on heart failure management: Global experience of the OPTIMIZE Heart Failure Care network. Int. J. Cardiol..

[59] Bader F., Manla Y., Atallah B., Starling R.C. (2021). Heart failure and COVID-19. Heart Fail. Rev..

[60] Meiler S.E., Ashton J.J., Moeschberger M.L., Unverferth D.V., Leier C.V. (1987). An analysis of the determinants of exercise performance in congestive heart failure. Am. Heart J..

[61] Quadagno D., Nation A.J., Johnson D., Waitley C., Waitley N., Epstein D., Satterwhite A. (1995). Cardiovascular disease and sexual functioning. Appl. Nurs. Res..

[62] Jaarsma T., Steinke E.E., Perk J., Gohlke H., Hellemans I., Sellier P., Mathes P., Monpère C., McGee H., Saner H. (2007). Sexual Counseling of the Cardiac Patient. Cardiovascular Prevention and Rehabilitation.

[63] Hoekstra T., Lesman-Leegte I., Couperus M.F., Sanderman R., Jaarsma T. (2012). What keeps nurses from the sexual counseling of patients with heart failure?. Heart Lung.

[64] Byrne M., Doherty S., Murphy A.W., McGee H.M., Jaarsma T. (2013). The CHARMS Study: Cardiac patients’ experiences of sexual problems following cardiac rehabilitation. Eur. J. Cardiovasc. Nurs..

[65] Doherty S., Byrne M., Murphy A.W., McGee H.M. (2011). Cardiac Rehabilitation Staff Views about Discussing Sexual Issues with Coronary Heart Disease Patients: A National Survey in Ireland. Eur. J. Cardiovasc. Nurs..

[66] Kolbe N., Kugler C., Schnepp W., Jaarsma T. (2016). Sexual Counseling in Patients with Heart Failure: A Silent Phenomenon: Results From a Convergent Parallel Mixed Method Study. J. Cardiovasc. Nurs..

[67] McMaughan D.J., Oloruntoba O., Smith M.L. (2020). Socioeconomic Status and Access to Healthcare: Interrelated Drivers for Healthy Aging. Front Public Health.

[68] Center for Substance Abuse Treatment (2014). SAMHSA/CSAT Treatment Improvement Protocols. Improving Cultural Competence.

[69] Kline K.S., Scott L.D., Britton A.S. (2007). The use of supportive-educative and mutual goal-setting strategies to improve self-management for patients with heart failure. Home Healthc. Nurse.

[70] Toback M., Clark N. (2017). Strategies to improve self-management in heart failure patients. Contemp. Nurse.

