# Supplementary material for: Inpatient vs. Outpatient: A Systematic Review of Information Needs throughout the Heart Failure Patient Journey
Source: J Clin Med. 2024 Feb 14;13(4):1085. doi: 10.3390/jcm13041085 (PMC10889710; doi:10.3390/jcm13041085)
Supplement: Supplementary file 1 [file jcm-13-01085-s001.zip › jcm-2861592-supplementary.pdf]

Supplementary File S1

Ghisi, Gabriele: Heart Failure & Information Need

Search Strategy: Ovid MEDLINE(R) ALL <1946 to February 15, 2023>

----

1 exp Heart Failure/ (144105)
2 ((heart or cardiac or myocardial) adj3 failure*).tw,kw. (216294)
3 (heart failure* adj3 patient*).tw,kw. (53464)
4 or/1-3 (255409)
5 patient education as topic/ (88189)
6 health education/ (63406)
7 Health Knowledge, Attitudes, Practice/ (125653)
8 exp consumer health information/ (12990)
9 exp "Surveys and Questionnaires"/ (1198239)
10 ((educat* or knowledg*) adj4 (need* or patient* or caregiver* or heart failure* or gap* or level)).tw,kw. (216934)
11 (information* adj4 (need* or heart failure* or acquisition)).tw,kw. (34357)
12 (patient* adj4 (aware* or unaware* or understand* or self-care or perception*)).tw,kw. (71369)
13 ((survey* or questionnaire* or tool*) adj4 (heart failure* or knowledg* or educat*)).tw,kw. (34650)
14 or/5-12 (1607693)
15 4 and 14 (17943)
16 limit 15 to "humans only (removes records about animals)" (17758)
17 16 not (Letter or Comment or Note).pt. (17236)

****
